# Spatial genetic structure and landscape connectivity in black bears: Investigating the significance of using different land cover datasets and classifications in landscape genetics analyses

**DOI:** 10.1002/ece3.7111

**Published:** 2021-01-05

**Authors:** Hope M. Draheim, Jennifer A. Moore, Scott R. Winterstein, Kim T. Scribner

**Affiliations:** ^1^ Department of Zoology Michigan State University East Lansing Michigan USA; ^2^ Biology Department Grand Valley State University Allendale Michigan USA; ^3^ Department of Fisheries and Wildlife Michigan State University East Lansing Michigan USA

**Keywords:** black bear, land cover data, landscape genetics, spatial genetic structure, *Ursus americanus*

## Abstract

Landscape genetic analyses allow detection of fine‐scale spatial genetic structure (SGS) and quantification of effects of landscape features on gene flow and connectivity. Typically, analyses require generation of resistance surfaces. These surfaces characteristically take the form of a grid with cells that are coded to represent the degree to which landscape or environmental features promote or inhibit animal movement. How accurately resistance surfaces predict association between the landscape and movement is determined in large part by (a) the landscape features used, (b) the resistance values assigned to features, and (c) how accurately resistance surfaces represent landscape permeability. Our objective was to evaluate the performance of resistance surfaces generated using two publicly available land cover datasets that varied in how accurately they represent the actual landscape. We genotyped 365 individuals from a large black bear population (*Ursus americanus*) in the Northern Lower Peninsula (NLP) of Michigan, USA at 12 microsatellite loci, and evaluated the relationship between gene flow and landscape features using two different land cover datasets. We investigated the relative importance of land cover classification and accuracy on landscape resistance model performance. We detected local spatial genetic structure in Michigan's NLP black bears and found roads and land cover were significantly correlated with genetic distance. We observed similarities in model performance when different land cover datasets were used despite 21% dissimilarity in classification between the two land cover datasets. However, we did find the performance of land cover models to predict genetic distance was dependent on the way the land cover was defined. Models in which land cover was finely defined (i.e., eight land cover classes) outperformed models where land cover was defined more coarsely (i.e., habitat/non‐habitat or forest/non‐forest). Our results show that landscape genetic researchers should carefully consider how land cover classification changes inference in landscape genetic studies.

## Graphical abstract

We evaluated the performance of multiple of resistance surfaces generated from two different land cover data sets to quantify the effects of landscape features on gene flow. We observe models with land cover defined with more complexity (i.e., eight land cover classes) out performed those models that defined land cover using a binary classification scheme (i.e., habitat/non‐habitat or forest/non‐forest.) Our analyses demonstrate that different land cover data sets classifications can result in disparate landscape–genetic associations.

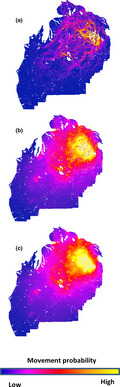

## 1 INTRODUCTION

Natural ecosystems are spatially heterogeneous and temporally dynamic. This complexity has important ecological implications for the organisms inhabiting these ecosystems and dispersing through them (Bolliger et al., 2010; Spear et al., 2010; Storfer et al., 2007). Identifying factors that influence the degree of population connectivity across heterogeneous landscapes provides insight into population dynamics and the evolutionary trajectory of a species (Segelbacher et al., 2010). For example, wide ranging species such as the American black bear (*Ursus americanus*) can be sensitive to land cover (Pelton, 1992). Fragmentation of preferred forest cover and increasing prevalence of human‐altered landscapes can impede or facilitate longer black bear dispersal (Cushman, 2006; Draheim et al., 2018; Short Bull et al., 2011). Landscape genetic approaches are valuable for understanding the effects of land use/land cover on species movements and abundance, and enable managers to target regions or habitat types that are important to maintain connectivity across anthropogenically altered habitats and ultimately assess the impacts of future change (Bolliger et al., 2010; Manel & Holderegger, 2013).

Traditional landscape genetic analyses often require generation of resistance surfaces. These surfaces typically take the form of a grid (raster) with cells (pixels) that are coded a priori to reflect predictions on the degree to which landscape or environmental features promote or inhibit animal gene flow (Spear et al., 2010). How accurately resistance surfaces predict the association between the landscape and movement is determined in large part by (a) the landscape features used in modeling, (b) the resistance (cell) values assigned to features and (c) how accurately resistance surfaces represent the actual landscape.

Expert opinion has been the most common way to assign resistance values. However, expert opinion has fallen out of favor over the recent years due to the subjective nature of this approach (Zeller et al., 2016). Simulation studies have found that landscape genetic associations are sensitive to parameterization of resistance weights (Koen et al., 2012). One way to improve resistance surface accuracy is to use a more rigorous analytical method such as habitat preference models or genetic data to assign biologically relevant weights (Cushman & Lewis, 2010; Peterman, 2018).

In spite of the analytical advancements in assigning biologically plausible resistance values, these approaches may not improve accuracy of predictions if there is uncertainty in the underlying datasets used for the parametrizations. For example, land cover is an indicator of ecological suitability (e.g., habitat and available resources) for a given species and a common predictor variable used in landscape genetic studies. Land cover resistance surfaces are typically constructed using publicly available land cover data layers (e.g., National Land Cover Database (NLCD), Global Land Cover (GLC) database, and NOAA’s Coastal Change Analysis Program (CCAP) (Bartholomé & Belward, 2005; Homer et al., 2007; NOAA, 2014). Often datasets are produced using different data sources, foci, algorithms and class definitions, and therefore, differ in their representation of the actual landscape (Kienast, 1993). Previous studies have shown disparities among land cover datasets, even those that are derived from the same satellite imagery (Foody, 2002; Ge et al., 2007; Pérez‐Hoyos et al., 2017; Tsendbazar et al., 2015). These disparities demonstrate the need to make informed decisions regarding which data layer to use or how fine (many classes) or coarse (few classes) land cover data are classified. The choice of a particular land cover dataset or classification scheme could ultimately impact downstream analytical outcomes and inferences that may be used to direct management decisions (Bai et al., 2014; Ge et al., 2007; Pérez‐Hoyos et al., 2017; Tsendbazar et al., 2015).

While many scientific articles have lauded the integration of GIS and genetic data (Bolliger et al., 2010; Spear et al., 2010; Storfer et al., 2007), few have provided guidance to practitioners about how freely available land cover data are generated or how classification accuracy (i.e., uncertainty) is assessed. Neither have studies routinely compared the degree of dissimilarity (i.e., classification inconsistences) among datasets. While a few studies have assessed performance of resistance surfaces by varying the parameters (i.e., cost values) to create resistance surfaces (Cushman & Landguth, 2010; Graves et al., 2012; Spear et al., 2010), comparatively less attention has been paid to understanding how the selection of different data layers that are meant to represent the same landscape features may impact the detection of associations between genetic and landscape variation.

Here we evaluate the performance of landscape genetic resistance models generated using two freely available land cover datasets. For each dataset, we created resistance surfaces using the same ecological model and biological assumptions then evaluated the influence of landscape features on spatial genetic structure of a large black bear population in Michigan's Northern Lower Peninsula (NLP), USA. Our three main objectives were (a) define fine‐scale spatial genetic structure, (b) identify landscape features that impede or facilitate gene flow, and (c) compare genetic and landscape associations estimated using different land cover datasets to understand the influence land cover dataset selection and reclassification have on landscape genetic analyses.

## 2 MATERIAL AND METHODS

### 2.1 Sampling, DNA extraction, PCR amplification

**Figure 1:**
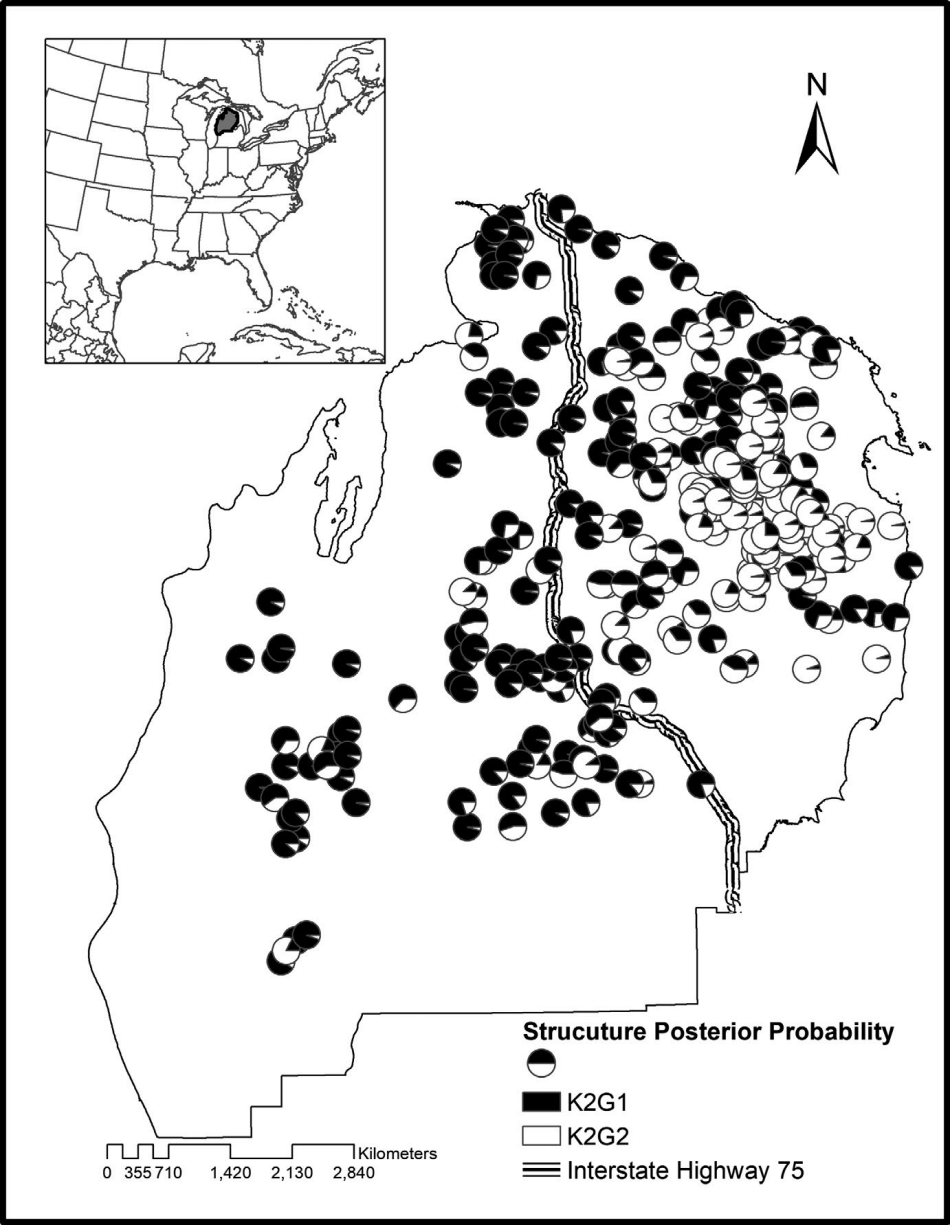
Study area in the Northern Lower Peninsula (NLP) of Michigan, USA showing locations of black bear harvest samples collected during 2006 (*N* = 365). Pie graphs were constructed using individual posterior probabilities of membership to each genetic cluster (*K* = 2) identified in Program STRUCTURE. K2G1 = genetic cluster one (western) and K2G2 = genetic cluster two (eastern)

Tissue samples (teeth, *N* = 365) were collected from harvested bears registered at hunter check stations during the 2006 fall harvest (September‐October) in Michigan's Northern Lower Peninsula (NLP, 47,739 km^2^; Figure 1). Bear harvest locations were recorded as township, range, and section. In Michigan, a survey township is a square unit of land containing approximately 36 square miles. Each square mile (2.59 km^2^) is a section. Harvest locations reported to a section were georeferenced using the section centroid and converted to UTM coordinates.

We extracted DNA from bear teeth using Qiagen DNEasy Tissue Kits (Qiagen Inc.) following manufacturer protocols. DNA was quantified using a Nanodrop spectrophotometer (Thermo Scientific) and diluted to a 20 ng/μl working concentration. We used PCR to amplify 12 variable microsatellite loci including: G10X, G10L, G10D, G10B, G10M (PCR annealing temperature *TA* = 58°C; Paetkau et al., 1995) UarMU59, UarMU50 (*TA* = 58°C, Taberlet et al., 1997), ABB1, ABB4 (*TA* = 54°C; Wu et al., 2010), UT29, UT35, and UT38 (*TA* = 54°C; Shih et al., 2009). We amplified DNA according to conditions outlined in Moore et al. (2014). Amplified products were sized on 6.5% denaturing acrylamide gels for electrophoresis and visualized on a LI‐COR 4,200 Global IR2 System (LI‐COR Inc.). All individual genotypes were scored independently by two experienced laboratory personnel using SAGA genotyping software (LI‐COR Inc.). To assess genotyping error, 10% of samples were randomly selected and genotyped a second time to yield a genotyping error rate of <2%.

### 2.2 Population genetic analysis

We tested for the presence of null alleles and allelic dropout using program MICRO‐CHECKER (Van Oosterhout et al., 2004). We tested for deviations from Hardy–Weinberg equilibrium using program GENEPOP (Version 3.1d; Raymond & Rousset, 1995), and used sequential Bonferroni tests to correct for multiple tests (Rice, 1989). We used Bonferroni corrections (Goudet, 1995, 2001) to test for linkage disequilibrium using program FSTAT 2.93. We quantified microsatellite genetic diversity and statistical power using mean number of alleles (*A*), observed heterozygosity (*H* _o_), and expected heterozygosity (*H* _e_) over all loci, and probability of identity (P_ID_) using program GenAlEx (Peakall & Smouse, 2006, v. 6.0).

We assessed spatial genetic structure using a global genetic spatial autocorrelation coefficient (*r*) calculated using program GenAlEx (Peakall & Smouse, 2006, v. 6.0). Coefficient *r* (range, −1 to + 1) was calculated by correlating pairwise geographical and inter‐individual genetic distance. We used program STUCTURE v. 2.3.4 (Pritchard et al., 2000) to characterize spatial genetic structure. We estimated the number of genetic clusters (*K*) without geographic location information and the posterior probability of each individual belonging to each cluster. We performed 10 independent runs of *K* = 1–10 using simulations of 2 x 10^6^ iterations after a burn‐in period of 5 x 10^5^ Markov Chain Monte Carlo (MCMC) iterations. The most likely number of clusters was determined by the log likelihood of *K* and the posterior probability of *K* (*P(K|X*)) as determined by the method described in Pritchard et al. (2000) and estimates of delta *K* (*Dk*) (Evanno et al., 2005) using program STRUCTURE HARVESTER v. 06.93 (Earl, 2012). To visualize the spatial distribution of the genetic clusters, we used ArcGIS 10.1 to plot posterior probability of cluster membership for each individual. To measure genetic differentiation among individuals, we calculated the proportion of shared alleles (Dps; Bowcock et al., 1994) for each pairwise combination of individuals using GenAlEx v. 6.0 (Peakall & Smouse, 2006).

### 2.3 Landscape genetic analysis

**Figure 2:**
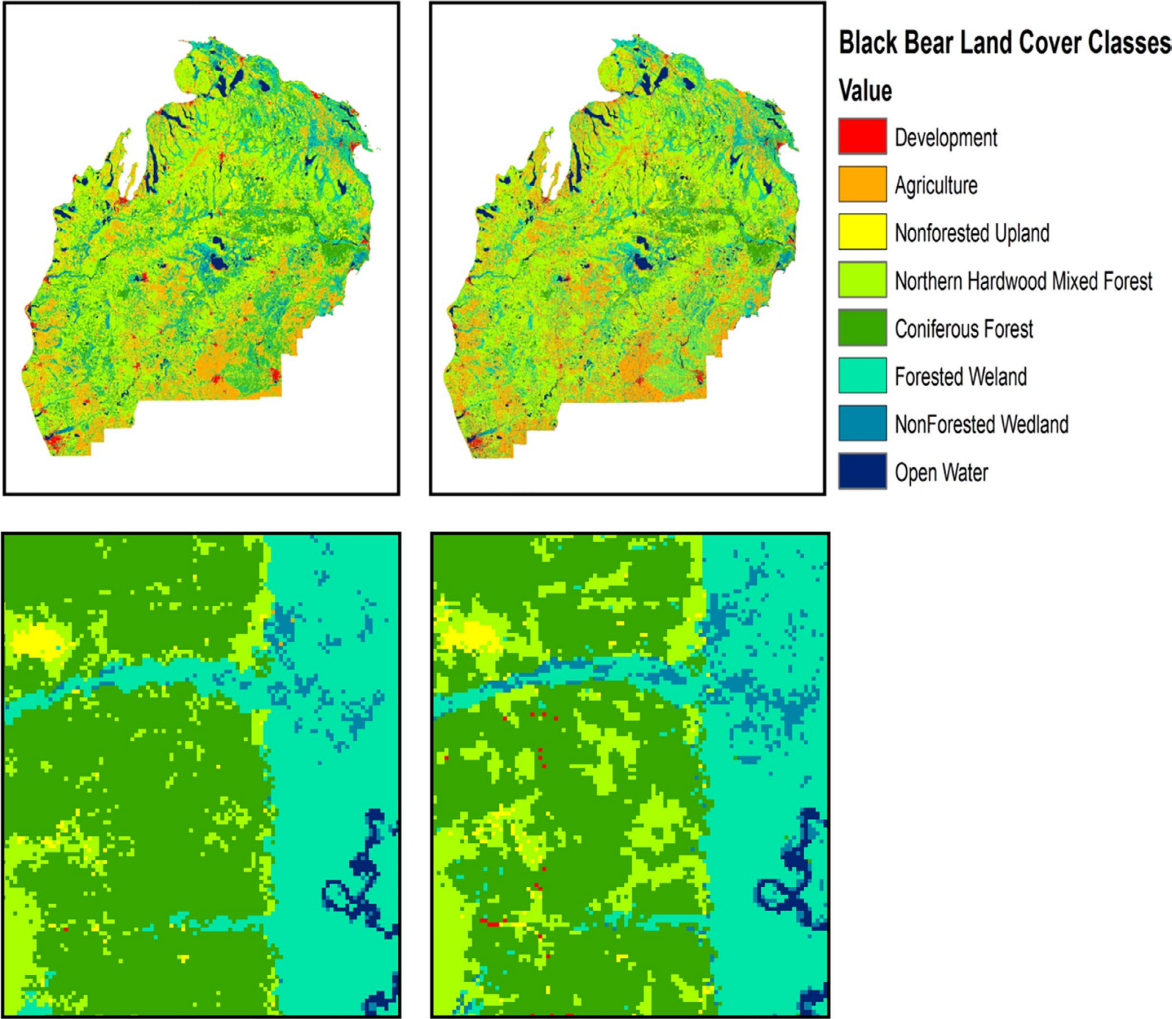
Side by side comparison of CCAP and IFMAP land cover maps using the black bear habitat suitability classification scheme described in Carter et al. (2010). (a) Land cover maps for the entire study area. (b) Land cover of the same 7 x 10 km area

We used two land use/land cover digital coverage maps derived from Landsat TM imagery; (a) Michigan Department of Natural Resources (MDNR) Resource Information Systems (MIRIS) Integrated Forest Monitoring, Assessment, and Prescription Project (IFMAP; MDNR, 2004) land cover data (resolution = 30 m), and (b) the National Oceanic and Atmospheric Administration (NOAA) Coastal Change Analysis Program (CCAP; NOAA, 2014) Land Cover data (resolution = 30 m). The IFMAP dataset is categorized into 34 land cover classes (Table S1). Reported accuracy assessment (based on 2,817 reference points) for IFMAP was 77% for overall accuracy and 68% for division among major forest types (i.e., mixed, deciduous, coniferous). MDNR defined a forest stand as coniferous, deciduous, or mixed using a 60% stand purity rule. That is, a forest stand is considered of mixed composition unless 60% of the forest stand is dominated by either coniferous or deciduous trees (MDNR, 2004). In contrast, the CCAP land cover dataset is classified into 25 land cover classes (Table S1) with an overall accuracy of 87% and 83% for major forest types based on 900 reference points. NOAA used a higher stand purity requirement (75%) for defining coniferous and deciduous forest types resulting in a larger proportion of forest stands either being classified as mixed forest or non‐forest (NOAA, 2014; Figure 2).

In order to understand how genetic differentiation was influenced by landscape features, we generated resistance surfaces using Spatial Analyst in ARCGIS 10.1. We chose each land cover feature according to positive or negative associations with black bear presence, based on habitat suitability estimates derived for NLP black bears by Carter et al. (2010). We generated a series of landscape resistance surfaces (i.e., models of landscape resistance to black bear gene flow) based on roads, rivers, and land cover, which have previously been reported to influence habitat selection by black bears in the NLP (Carter et al., 2010). We made the first set of resistance surfaces using a fine‐scale land cover classification scheme for the IFMAP or the CCAP datasets (resolution = 150 m). We reclassified the IFMAP land cover data according to bear habitat suitability into eight classes: northern hardwood mixed forest (comprised of northern hardwood, aspen, oak, upland hardwoods), forested wetland (lowland forest), coniferous forest (pine, conifers), non‐forested upland, agriculture, non‐forested wetland, open water, and developed (Table S1). We similarly reclassified the CCAP dataset based on bear habitat suitability: mixed/deciduous, forested wetland, coniferous forest (evergreen forest), non‐forested upland, agriculture, non‐forested wetland, open water, and developed (Table S1).

Following reclassification of our land cover datasets using Carter et al. (2010), we generated a confusion matrix (error matrix, Foody, 2002) to compare the concordance of pixels (i.e., raster cells) between the CCAP and IFMAP datasets. In other words, for a given pixel, we quantified how often the two land cover datasets assigned the same land cover class, and how often pixels were classified differently. Our confusion matrix is a *c* x *c* (c = is the number land cover classes) table that quantifies the agreement (diagonal elements) and disagreement (off‐diagonal elements) of land cover classification between the CCAP and IFMAP land cover datasets. In our matrix, the columns of Table 1 represent the assigned class of each pixel according to the CCAP land cover dataset. The rows are the class of the same pixels according to the IFMAP land cover dataset. The cells of the table therefore show the number of pixels of a particular class (i.e., developed) that is classified as either the same class or any alternative class between the two datasets (Table 1).

**Table 1:** Confusion matrix for evaluating agreement and disagreement of land cover classification between IFMAP and CCAP land cover datasets (resolution 150 m)

IFMAP Classes	CCAP Classes							
	Developed	Agriculture	Non‐forested upland	Mixed/ Deciduous Forest	Evergreen Forest	Forested Wetland	Non‐forested Wetland	Open Water
Developed	13,967	7,071	7,764	3,218	1699	507	736	233
Agriculture	3,604	96,513	19,223	4,760	1,091	754	918	29
Non‐forested upland	2,810	4,932	64,606	19,218	6,172	1,238	1,491	53
Mixed/Deciduous Forest	3,274	2,922	24,487	231,364	8,075	7,380	3,115	135
Evergreen Forest	889	555	5,836	16,032	51,778	2,632	860	123
Forested Wetland	707	598	3,311	12,928	4,380	61,703	13,458	294
Non‐forested Wetland	478	481	2,906	3,735	1,002	14,034	24,428	809
Open Water	88	112	106	206	134	231	890	24,749
Total	25,816	113,183	128,239	291,460	74,330	88,479	45,895	26,425
Class Changes	11,849	16,670	63,632	60,096	22,553	26,775	21,467	1676
Percent Similarity	54%	85%	50%	79%	70%	70%	53%	94%

Note

The diagonal elements are the number of pixels where land cover classifications agree, off‐diagonal elements are the number of pixels where classifications disagree (Total pixels = 793,826). The columns represent what class is assigned to each pixel by the CCAP dataset and the rows are what the same pixels are according to the IFMAP dataset.

We also modeled resistance as a function of land cover according to two alternative hypotheses, using a binary classification of land cover: (a) bear habitat (significantly positively correlated with bear presence; IFMAP = northern hardwood mix, aspen, forested wetland; CCAP = mixed/deciduous, forested wetland) or non‐habitat (either positively associated but not significant or significantly negatively correlated with bear presence; IFMAP = pine, oak, non‐forested upland, agriculture, non‐forested wetland, developed; CCAP = evergreen forest, non‐forested upland, agriculture, non‐forested wetland, developed), and (b) forested areas (IFMAP = northern hardwood mix, aspen, forested wetland, pine, oak; CCAP = mixed forest/deciduous, forested wetland, evergreen forest) or non‐forested areas (IFMAP = non‐forested uplandagriculture, non‐forested wetland, developed; CCAP = non‐forested upland, agriculture, non‐forested wetland, developed). Major rivers and all roads (Interstate‐75, state roads, all other roads) were included as predictor variables as potential physical barriers to dispersal. We proposed 18 alternative landscape hypotheses (Table 2).

**Table 2:** The highest ranked a priori landscape models and the null models explaining black bear genetic distance

Rank	Model	LL	k	AIC*_c_*	∆AIC*_c_*	*w_i_*	*R* ^2^
1	Roads + River	−140,596.39	7	281,200.78	0.00	0.55	0.063
2	Interstate 75 only	−140,597.20	3	281,202.40	1.62	0.24	0.055
3	Roads	−140,597.35	5	281,202.71	1.93	0.21	0.052
4	CCAP HSI land cover only	−140,640.89	9	281,289.78	89.00	0	0.048
5	IFMAP HSI land cover only	−140,641.67	9	281,291.35	90.57	0	0.040
6	CCAP Hab/Non‐Hab land cover only	−140,653.36	3	281,314.73	113.95	0	0.024
7	IFMAP Hab/Non‐Hab land cover only	−140,654.67	5	281,317.33	116.55	0	0.001
8	Rivers	−140,655.00	3	281,318.30	117.52	0	0.022
9	CCAP Forest/Non Forest land cover	−140,655.70	3	281,319.40	118.63	0	0.022
10	IFMAP Forest/Non Forest land cover only	−140,655.96	3	281,319.93	119.15	0	0.017
11	Null	−140,656.19	1	281,320.38	119.60	0	
12	Distance	−140,735.54	2	281,477.08	276.31	0	0.044
13	IFMAP HSI + Rivers	−140,735.26	11	281,478.51	277.73	0	−0.053
14	CCAP HSI + Rivers	−140,735.32	11	281,478.63	277.85	0	−0.062
15	CCAP HSI + Roads +River	−140,735.41	17	281,478.83	278.05	0	−0.063
16	IFMAP HSI + Roads +River	−140,735.53	17	281,479.05	278.27	0	−0.054
17	CCAP HSI + Roads	−140,735.54	13	281,479.07	278.29	0	−0.021
18	IFMAP HSI + Roads	−140,735.54	13	281,480.56	279.78	0	−0.021

Note

∆AIC*_c_*, the difference between AIC*_c_* for the alternative model compared to the highest ranked model; AIC*_c_*, the AIC*_c_* score; CCAP, Coastal Change Analysis Program land cover dataset; Hab, Habitat; HSI, Habitat Suitability Index, designating models in which land cover is reclassified using Carter et al., 2010 ecological model; IFMAP, Integrated Forest Monitoring, Assessment, and Prescription Project land cover dataset; k, number of parameters in the model; LL, log likelihood; Model, name of the model; *R* ^2^, *r* squared; Rank, the rank based on AICc values; *w_i_*, Akaike's weight.

The models are ranked based on the smallest AIC*_c_* value.

To eliminate the subjectivity in assigning resistance weights to resistance surfaces, we used a genetic algorithm implemented in the R‐package ResistanceGA (Peterman, 2018) to parameterize individual resistance values for a give resistance surface based on pairwise genetic distance. The optimized surfaces are generated using sample locations and effective distances calculated by CIRCUITSCAPE v. 4.0 (McRae, 2006) to maximize the fit of a resistance model to the genetic dataset using linear mixed effects models and AIC*_c_* values (Peterman, 2018). Optimization proceeded until no further improvement of AIC*_c_* was achieved. For our analysis, our input landscape surfaces incorporated one or more landscape variables including land cover, rivers, and roads (predictor variables were not correlated (|*r*| < .33). We randomly selected 340 samples to meet the pre‐condition of the program, which only allows one genetic sample per surface pixel. We used the proportion of shared alleles between all pairs of individuals (Dps; Bowcock et al., 1994) as the dependent variable. We ran each model twice as recommended by Peterman (2018) to evaluate output for consistency between replicate runs. Model testing was performed by fitting a maximum‐likelihood population effects (Clarke et al., 2002) model to relate genetic distance (Dps) to resistance distance for each candidate model. In addition to landscape resistance surfaces, we assessed Euclidean distance alone (isolation by distance, IBD) as well as an intercept only null model. Model selection was determined by comparison of AIC*_c_* and Akaike weight (*w_i_*) following Burnham and Anderson (2002).

## 3 RESULTS

### 3.1 Population genetics

We found no evidence for null alleles or allelic dropout, and no loci were found to deviate significantly from Hardy–Weinberg or linkage equilibrium, so all 12 loci were retained for further analyses. Across all loci, expected heterozygosity ranged from 0.67 to 0.91, number of alleles per locus ranged from six to 26 (Table S2), and P_ID_ was 2.2 x 10^–14^.

**Figure 3:**
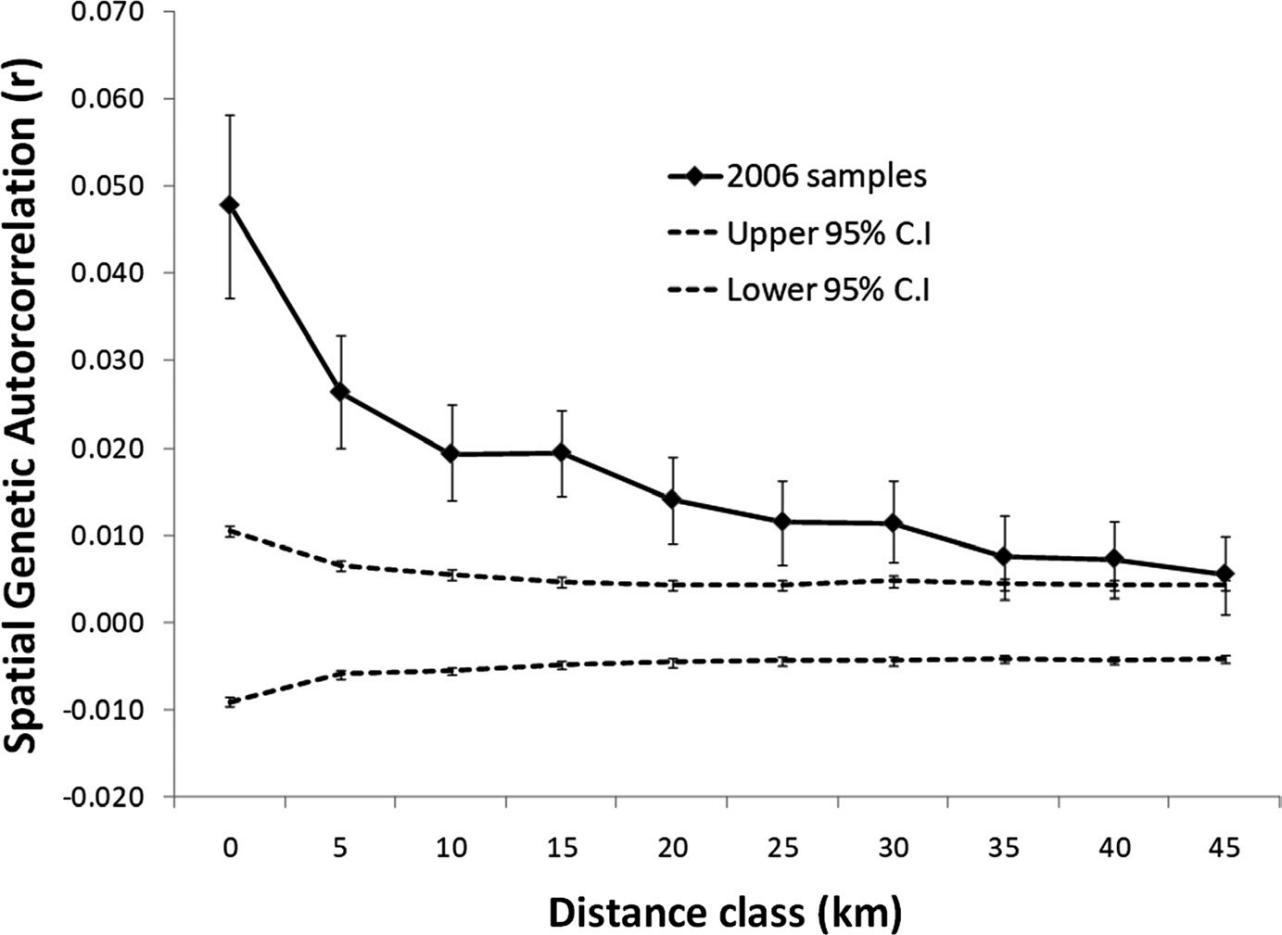
Global spatial autocorrelation of black bear samples. Bars represent standard errors. The 95% confidence intervals (dashed lines) about the null hypothesis of no spatial structure were estimated by permutations based on distance classes of 5 km

The NLP black bear population is not genetically homogeneous. Global spatial autocorrelation analysis revealed an isolation by distance pattern, whereby genetically similar individuals were not randomly distributed. Individuals sampled at inter‐individual distances of 0–30 km showed significant positive spatial genetic autocorrelation (Figure 3). Our STRUCTURE results indicated the highest average log‐likelihood value (−15,714.50) was observed for *K* = 4. However, using Delta *K*, as recommended by Evanno et al. (2005), the greatest support was for *K* = 2, which suggest the NLP bears consist of two genetic clusters. Distribution of individual posterior probabilities of cluster membership indicated a longitudinal (east‐west) gradient (Figure 1).

### 3.2 Landscape genetic analysis

Quantitative comparison of the IFMAP and CCAP land cover datasets revealed 21% of pixels were classified differently between the two datasets (Table 1). Most notably, pixels classified as developed, non‐forested upland, and non‐forested wetland in the CCAP dataset were most frequently classified as a different land cover class for the IFMAP dataset.

Landscape resistance modeling revealed the best univariate models based on AIC*_c_* included: (a) interstate 75 only, (b) roads only, and (c) land cover only (for both IFMAP and CCAP datasets) classified according to the habitat suitability model from Carter et al. (2010). All three outperformed either the distance only or null model. However, when multiple landscape predictors were considered, the best performing model included rivers and roads (Akaike weight, w_i_ = 0.55) indicating rivers + roads, in the absence of land cover, were good predictors of gene flow (Table 2). In spite of the 21% discordance in pixel classification between the two land cover datasets, we found no differences in model performance among the models using the different land cover datasets. However, we did observe models with land cover defined with more complexity (i.e., eight land cover classes) outperformed those models that defined land cover using a binary classification scheme (i.e., habitat/non‐habitat or forest/non‐forest.)

## 4 DISCUSSION

Comparative studies are needed to assess different landscape genetic approaches. As we have shown, differences do exist among land cover datasets and have the potential to affect landscape genetic inference, yet effects are not often considered in landscape genetic studies. Our study centered on comparing the effects of using two different land cover datasets generated using different criteria to infer landscape‐genetic associations in a large bear population in Michigan's NLP, USA. We found that landscape factors better predicted black bear genetic differentiation than distance alone and found no differences in model performance between land cover datasets, despite marked dissimilarity in land cover classifications.

### 4.1 Land cover dataset comparison

Comparisons among land cover datasets revealed that 21% of the pixels differed in their land cover classification between the IFMAP and CCAP datasets even though the datasets were generated using the same satellite imagery (Table 1, Figure 2a). Differences may be attributed to several factors. First, despite the potential for high‐resolution satellite imagery to pick up fine‐scale differences in land cover, no classification is ever a perfect representation of the landscape. Digitized land cover maps are models or generalizations; thus, they have inherent limitations. Uncertainty or error in remote sensing is primarily associated with land cover class misidentification (Foody, 2002; Liu et al., 2004). Land cover classification can be subjective (Thomlinson et al., 1999) and can have a propensity for high error rates, despite recommended guidelines to ensure accuracy of classifications (e.g., overall accuracy of 85% with no class less than 70% accurate; Congalton & Green, 2008). For example, the same satellite images classified by two independent researchers, but applying the same definition of land cover types, can produce different land cover maps. The IFMAP and CCAP datasets differed considerably in overall misclassifications (e.g., accuracy assessments; IFMAP > 77%, CCAP > 87%), and propensity for misclassification increases when examining closely related land cover types (e.g., accuracy assessment among forest types; IFMAP > 67%, CCAP > 83%; Beier et al., 2008; MDNR, 2004; NOAA, 2014). Second, the data layers we used in our evaluation use different thresholds for stand dominance (IFMAP = 60%, CCAP = 75%) when classifying coniferous/deciduous forest cover types (MDNR, 2004). For the IFMAP data, the MDNR lowered the thresholds to 60% because much of Michigan's forests are mixed and agency personnel wanted to emphasize species predominance in cover type for forestry practices, thereby reducing the number of forest cover types that fall within mixed forest classes (MDNR, 2004). In addition, a lower stand threshold in the IFMAP data would result in more pixels identified as forested compared to the CCAP data. For example, 29% of cells classified as forested wetland in the IFMAP dataset are identified as non‐forested wetland in the CCAP dataset. Finally, the IFMAP dataset was published in 2001, five years before the CCAP data (year of publication 2006) and within the NLP there has been landscape modification from ongoing anthropogenic activities (primarily deforestation).

Surprisingly, despite the discrepancies observed among land cover datasets, we found that the source of the land cover data did not influence downstream analysis. That is, IFMAP and CCAP land cover were equally predictive of black bear gene flow. How a species responds to the landscape is in large part determined by a species dispersal potential and sensitivity to landscape configuration and complexity. Black bears are habitat generalists and exhibit large dispersal movements (Brodeur et al., 2008; Mitchell & Powell, 2007; Moore et al., 2014; Noyce & Garshelis, 2011; Rogers, 1987), thus have the potential to move efficiently through non‐preferred habitat. If broad scale spatial patterns (as opposed to fine/pixel scale) are more likely to influence black bear dispersal, it is not surprising that changes in class designations at a pixel scale may matter little compared to the scale at which bears perceive the landscape. Our findings of concordance between land cover datasets can only be applied to Michigan black bears in the NLP. Fine‐scale differences between land cover datasets may be more impactful for landscape genetic models of habitat specialists, less mobile species, or species that are particular sensitive to landscape modification (i.e., development, agriculture). Further investigations, for example using other land cover datasets, locales, and species, are needed to address this question.

### 4.2 Effect of landscape features on black bear connectivity

Black bears in the NLP exhibited significant positive spatial genetic autocorrelation for distances up to 30 km, consistent with IBD (Coulon et al., 2004; Wright, 1943). Positive spatial genetic autocorrelation over short distances is likely attributed to male‐biased dispersal and female natal philopatry, commonly exhibited by black bears, where female offspring establish home ranges adjacent to the mothers whereas male offspring disperse from the natal area (Costello, 2010; Moore et al., 2014; Rogers, 1977, 1987; Schwartz & Franzmann, 1992). Indeed, black bear dispersal in Michigan's NLP is strongly male biased (Moore et al., 2014; Waples et al., 2018). IBD is consistent with studies on bears and other wide‐ranging carnivores (Brown et al., 2009; Paetkau et al., 1997; Rueness et al., 2003).

We did, however, find isolation by landscape resistance to be more strongly supported than IBD and the null model. Our results using univariate landscape models suggest that roads were the landscape feature most strongly correlated with genetic distance for black bears in the NLP. We found state roads and interstate‐75 had a 600 to 1,000 times higher resistance value than low traffic paved/forest roads suggesting strong avoidance of crossing highways than other roads. In Michigan, hunting and vehicle collisions are the most common causes of recorded bear mortality (Frawley, 2010). McFidden‐Hiller et al. (2016) found increased human–bear interactions with high primary road (e.g., interstates, highways, and residential) density. Roads can negatively impact bears survival and connectivity by (a) providing ease of access for hunters, and (b) acting as a barrier to dispersal. Roads as dispersal barriers have been shown for both black bears (Lee & Vaughan, 2003; Thompson et al., 2005) and brown bears (Proctor et al., 2005) which is likely due to habitat disturbance or risk of vehicle collisions (McFadden‐Hiller et al., 2016). In addition, our STRUCTURE results indicate that two genetically distinct groups exist in the NLP, defined as a western and an eastern genetic cluster. Interestingly, running proximal to the primary shift from western to eastern individuals is Interstate‐75 (Figure 1) further indicating primary roads are barriers to gene flow for black bears in Michigan's NLP.

Comparison of land cover only models reveals land cover classified using the habitat suitability models from Carter et al. (2010) (Table 2) outperformed all models where land cover was classified using two coarse definitions; (a) habitat/non‐habitat or (b) forest/non‐forest. Previous studies have found strong support for associations between black bear genetic structure and land cover classified broadly as forest/non‐forest (Cushman et al., 2006; Short Bull et al., 2011). One possible explanation for a poorer model performance when land cover is coarsely defined in our study is the difference in spatial distribution of food availability between our study area and previous studies (i.e., NLP versus. Rocky Mountains). Unlike conifer‐dominated forest types that occur in the Rocky Mountains of the United States, Michigan's NLP forests are a heterogeneous mix of deciduous, coniferous, and mixed stands with different understory communities and thus food availabilities.

Comparisons between competing landscape models parameterized using single and multiple variables found that inclusion of more than a single landscape predictor improved model performance when rivers and roads were included. This result indicates that rivers may act as a semipermeable barrier to black bear gene flow; however, inclusion of rivers only slightly improved model performance (*R* ^2^ = .063) when compared to roads alone (i.e., I75 only = ΔAIC*_c_* > 1.62, *R* ^2^ = .055 or roads ΔAIC*_c_* > 1.93, *R* ^2^ = .052). Interestingly, when adding land cover classified based on the HSI to roads resulted in poorer performing models despite land cover only models performing better than the distance and null models (e.g., CCAP, ΔAIC*_c_* ~ 89, *R* ^2^ = .048; IFMAP, ΔAIC*_c_* = 90, *R* ^2^ = .040). One possible explanation is that land cover may not be a good predictor of gene flow overall. Thus, adding land cover to the model may have increased noise.

**Figure 4:**
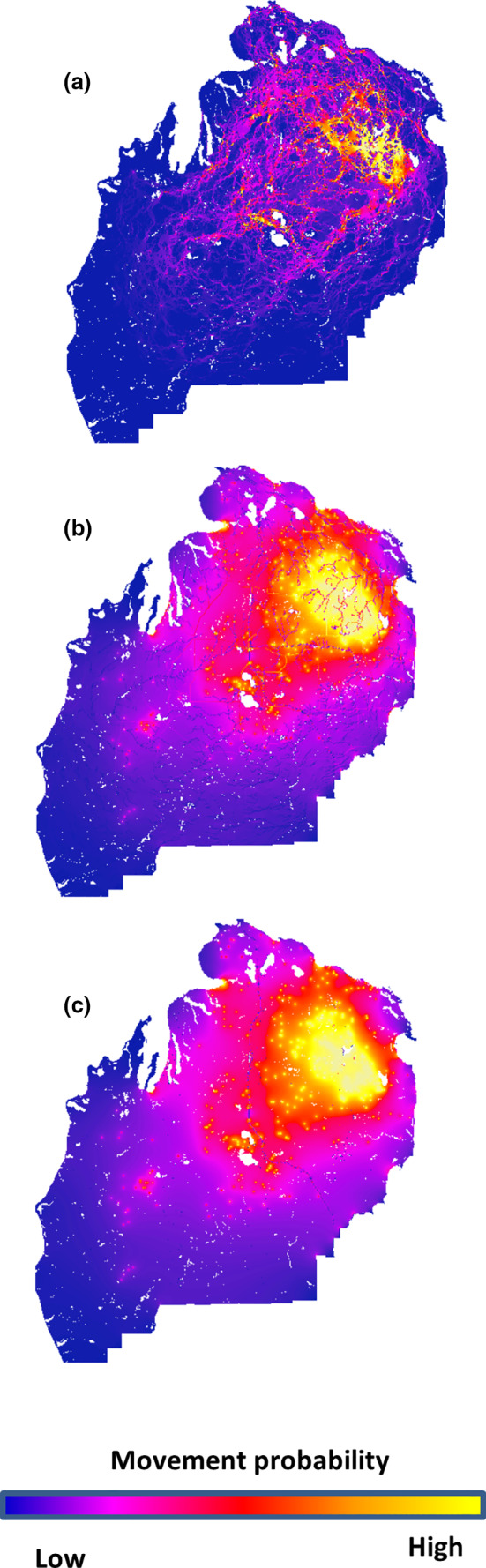
Comparison of cumulative resistance maps representing movement probability among pairs of individuals from three competitive isolation‐by‐resistance models (Table 2) based on (1) land cover only, generated from CCAP land cover; (2) roads and rivers; and (3) Interstate‐75 only using program CIRCUITSCAPE. Gradients of colors indicate the probability of black bear movement. Yellow colors indicate high probability; red and pink colors indicate medium probability; and purple and blue representing low probability

Visualization of potential dispersal pathways based on three models revealed similar patterns of high, medium, and low probability of bear movement (Figure 4). Heat maps show two clear areas of high flow; a large patch located in the east central region and a smaller patch located in the south central region of the study area (near Houghton Lake and Higgins Lake). Connecting these two regions are many areas of medium permeability suggesting dispersal within the NLP can occur via multiple pathways. Corridors, if adequate in arrangement and number, can in theory offset the negative consequences of landscape fragmentation and are necessary to maintain occupancy in a population that exhibits source‐sink dynamics (Dixo et al., 2009). Draheim et al. (2016) found black bears exhibit asymmetric dispersal among areas within the NLP suggesting loss of movement corridors could have wide ranging effects. For example, long‐term persistence of black bears in sink areas depends on the rate of extinction and the rate of movement between patches (Fahrig & Merriam, 1994). Thus, loss of corridors by habitat alteration in the NLP can decrease probability of local black bear occupancy.

## 5 CONCLUSION

Our results indicate considerable classification discrepancies between land cover datasets did not impact our landscape genetic results; however, land cover had a minimal influence on black bear connectivity compared to roads and rivers. We did find that how coarse (e.g., forest/non‐forest) or fine (e.g., classified using eight classes as identified by Carter et al., (2010)) land cover is defined before parametrization changed our understanding of how land cover affects black bear genetic connectivity in Michigan's NLP. Our ability to relate genetic distance to landscape elements is largely dependent on hypothesized parameterized resistance surfaces. However, resistance surfaces are also dependent on the landscape data layers used for parametrization. Indeed, we obtained varying degrees of genetic and landscape associations using the same landscape data, which differed only in classification complexity (i.e., many or few land cover types). We recommend that landscape genetic researchers should not only carefully consider which landscape variables they use and how they assign resistance weights (e.g., parametrization versus expert opinion) to generate resistance surfaces, but also scrutinize how landscape elements, such as land cover, are initially characterized.

## CONFLICT OF INTEREST

None declared.

## AUTHOR CONTRIBUTION

Hope Draheim: Conceptualization (lead); Data curation (lead); Formal analysis (lead); Funding acquisition (supporting); Investigation (lead); Methodology (lead); Project administration (lead); Writing‐original draft (lead); Writing‐review & editing (lead). Jennifer A Moore: Conceptualization (equal); Data curation (equal); Writing‐review & editing (supporting). Scott Winterstein: Funding acquisition (equal); Writing‐review & editing (equal). Kim Scribner: Conceptualization (supporting); Funding acquisition (lead); Supervision (equal); Writing‐review & editing (supporting).

## Supporting information

Supplementary Material

## Data Availability

Data used in this study, including bear microsatellite genotypes and harvest locations, are available for download on Dryad (https://doi.org/10.5061/dryad.c61q0).
