# Supplementary material for: Spatial genetic structure and landscape connectivity in black bears: Investigating the significance of using different land cover datasets and classifications in landscape genetics analyses
Source: Ecol Evol. 2021 Jan 5;11(2):978–89. doi: 10.1002/ece3.7111 (PMC7820153; doi:10.1002/ece3.7111)
Supplement: Supplementary file 1 — Supplementary Material [file ECE3-11-978-s001.docx]

**Supplemental Information**

Table S1. Land cover types according to IFMAP and CCAP classification schemes. Class number and description for the IFMAP and CCAP data sets were pulled from original data sets ([MDNR, 2004](#_ENREF_37), [NOAA, 2014](#_ENREF_40)). Class numbers and descriptions for the black bear ecological model were originated from Carter 2007.

|  |  |  |  |  |  |  | | |  |
| --- | --- | --- | --- | --- | --- | --- | --- | --- | --- |
|  |  |  |  |  |  |  | | |  |
| IFMAP | |  | CCAP | |  | Black Bear Ecological Model | | | |
|  |  |  |  |  |  |  | |  | |
|  |  |  |  |  |  |  | |  | |
| Class Description | |  | Class Description | |  | Class Description | | | |
|  |  |  |  |  |  |  |  | | |
| 110 | Low Intensity Urban |  | 3 | Low-Intensity Developed |  | 1 | Human Development | | |
| 123 | High Intensity Urban |  | 2 | High-Intensity Developed |  | 1 | Human Development | | |
| 121 | Airports |  | 2 | High-Intensity Developed |  | 1 | Human Development | | |
| 122 | Roads / Paved |  | 2 | High-Intensity Developed |  | 1 | Human Development | | |
| 2111 | Non-vegetated Agriculture |  | 20 | Bare Land |  | 2 | Agriculture | | |
| 2112 | Row Crops |  | 6 | Cultivated Land |  | 2 | Agriculture | | |
| 2113 | Forage / Pasture |  | 6 | Cultivated Land |  | 2 | Agriculture | | |
| 222 | Orchards / Vineyards / Nursery |  | 6 | Cultivated Land |  | 2 | Agriculture | | |
| 310 | Herbaceous Openland |  | 8 | Grassland |  | 3 | Upland Nonforested | | |
| 320 | Upland Shrub |  | 12 | Scrub/Shrub |  | 3 | Upland Nonforested | | |
| 330 | Low-Density Trees |  | 12 | Scrub/Shrub |  | 3 | Upland Nonforested | | |
| 350 | Parks / Golf Courses |  | 8 | Grassland |  | 1 | Upland Nonforested | | |
| 411 | Northern Hardwoods |  | 9 | Deciduous Forest |  | 4 | Northern Hardwood and Mixed Forest | | |
| 412 | Oak Type |  | 9 | Deciduous Forest |  | 5 | Oak Forest | | |
| 413 | Aspen Type |  | 9 | Deciduous Forest |  | 6 | Aspen Forest | | |
| 414 | Other Upland Hardwoods |  | 9 | Deciduous Forest |  | 4 | Northern Hardwood and Mixed Forest | | |
| 419 | Mixed Upland Deciduous |  | 9 | Deciduous Forest |  | 4 | Northern Hardwood and Mixed Forest | | |
| 421 | Pines |  | 10 | Evergreen Forest |  | 7 | Pine | | |
| 423 | Other Upland Conifers |  | 10 | Evergreen Forest |  | 7 | Pine | | |
| 429 | Mixed Upland Conifers |  | 10 | Evergreen Forest |  | 7 | Pine | | |
|  | |  |  |  |  |  |  | | |
| Table S1 (cont’d) | |  |  |  |  |  |  | | |
|  |  |  |  |  |  |  |  | | |
| 431 | Upland Mixed Forest |  | 11 | Mixed Forest |  | 4 | Northern Hardwood and Mixed Forest | | |
| 500 | Water |  | 21 | Water |  | 10 | Water | | |
| 611 | Lowland Deciduous Forest |  | 13 | Palustrine Forested Wetland |  | 8 | Forested Wetland | | |
| 612 | Lowland Coniferous Forest |  | 13 | Palustrine Forested Wetland |  | 8 | Forested Wetland | | |
| 613 | Lowland Mixed Forest |  | 13 | Palustrine Forested Wetland |  | 8 | Forested Wetland | | |
| 621 | Floating Aquatic |  | 22 | Palustrine Aquatic Bed |  | 9 | Nonforested Wetland | | |
| 622 | Lowland Shrub |  | 14 | Palustrine Shrub/Scrub Wetland |  | 9 | Nonforested Wetland | | |
| 623 | Emergent Wetland |  | 15 | Palustrine Emergent Wetland |  | 9 | Nonforested Wetland | | |
| 629 | Mixed Non-Forest Wetland |  | 15 | Palustrine Emergent Wetland |  | 9 | Nonforested Wetland | | |
| 710 | Sand / Soil |  | 20 | Bare Land |  | 2 | Agriculture | | |
| 720 | Exposed Rock |  | 20 | Bare Land |  | 2 | Agriculture | | |
| 730 | Mud Flats |  | 19 | Unconsolidated Shore |  | 2 | Agriculture | | |
| 790 | Other Bare /Sparsely Vegetated |  | 20 | Bare Land |  | 2 | Agriculture | | |
|  |  |  |  |  |  |  |  | | |

CCAP = Costal Change Analysis Program land cover dataset.

IFMAP = Integrated Forest Monitoring and Assessment Prescription Project land cover dataset.

Table S2. Black bear genetic diversity at 12 microsatellite loci. No loci showed significant deviations from Hardy-Weinberg equilibrium ([Raymond and Rousset, 1995](#_ENREF_50)). *Na*, number of alleles per locus; *Ho*, observed heterozygosity; *H_E_*, expected heterozygosity.

|  |  |  |  |
| --- | --- | --- | --- |
| **2006** | | | |
| **Locus** | ***Na*** | ***H_O_*** | ***H_E_*** |
|  |  |  |  |
| X | 13 | 0.672 | 0.696 |
| M | 9 | 0.789 | 0.780 |
| D | 9 | 0.765 | 0.769 |
| L | 10 | 0.827 | 0.854 |
| B | 6 | 0.678 | 0.717 |
| Uar50 | 9 | 0.669 | 0.769 |
| Uar59 | 7 | 0.642 | 0.671 |
| ABB1 | 9 | 0.693 | 0.729 |
| ABB4 | 8 | 0.788 | 0.799 |
| UT35 | 6 | 0.774 | 0.784 |
| UT38 | 26 | 0.893 | 0.909 |
| UT29 | 12 | 0.871 | 0.826 |
|  |  |  |  |
|  |  |  |  |

**REFERENCE**

Carter, N. H. (2007). Predicting ecological and social suitability of black bear habitat in Michigan's Lower Peninsula. Master of Science: University of Michigan.
